# Coronal Knee Alignment 40 Years after Total Meniscectomy in Adolescents: A Prospective Cohort Study

**DOI:** 10.2174/1874325001711010424

**Published:** 2017-05-30

**Authors:** I.P. Pengas, W. Nash, Wasim Khan, A. Assiotis, J. Banks, M.J. McNicholas

**Affiliations:** 1 University Hospitals of Coventry & Warwickshire, Clifford Bridge Road, Coventry, West Midlands, CV2 2DX, UK; 2 Queen Elizabeth Hospital, Stadium Road, Woolwich, London, SE18 4QH, UK; 3 Cardiff and Vale Orthopaedic Centre, Llandough Hospital, University Hospital Wales Health Board, Cardiff, CF64 2XX, UK; 4 Hillingdon Hospital, Pield Heath Road, Uxbridge. UB8 3NN, UK; 5 University Hospital Aintree Teaching Hospital NHS Foundation Trust, Longmoor Lane, Liverpool, L9 7AL, UK

**Keywords:** Meniscectomy, Malalignment, Radiographic scoring, Patient reported outcome measures

## Abstract

**Introduction:**

Meniscectomies result in altered knee biomechanics and increase contact forces on the operated knee joint.

**Methods:**

We assessed coronal knee alignment in relation to radiological osteoarthritis grading, clinical range of movement and patient reported outcome measures 40 years after total open meniscectomies in adolescence. Thirty eight knees (30 patients) that underwent total open meniscectomy were assessed on standardised weight-bearing anteroposterior radiographs for deviation from ‘physiological valgus angle’ in either direction (magnitude of malalignment). These values were analysed as per site of meniscectomy for correlations with radiographic scoring systems, range of motion and patient reported outcome measures.

**Results:**

Tibiofemoral angle was significantly more varus, and the magnitude of malalignment was significantly higher for the medial meniscectomy patients. The range of flexion was lower for those patients who underwent medial and lateral meniscectomies of the same knee. The patients who underwent meniscectomies of both knees had worse scores for IKDC and KOOS quality of life. Tibiofemoral angle, magnitude of malalignment and range of flexion strongly correlated with Ahlback, and Kellgren and Laurence scores, but patient reported that outcome measures did not correlate.

**Conclusion:**

Meniscectomy induced malalignment corresponds to the site of meniscectomy and the radiographic degree of osteoarthritis. While malalignment and reduced range of movement correlate well with worsening radiographic signs of arthritis, patient reported outcome measures do not correlate.

## INTRODUCTION

Total meniscectomy historically was advocated for even insignificant meniscal pathology as it was believed that the removal of the meniscus would result in a fibrous replica of the original tissue [1-3]. Fairbank was the first to document radiographic changes consistent with osteoarthritis following such procedure [4]. We have previously shown, in the same cohort of patients included in this study, that meniscectomy leads to symptomatic osteoarthritis of the knee and an increased rate of total knee replacement [5]. Now, total meniscectomy is associated with radiographic progression of osteoarthritis and subsequent deterioration in long-term functional outcomes [6].

In the coronal plane, the normal Lateral Distal Femoral Angle (LDFA) of 81º and Medial Proximal Tibial Angle (MPTA) of 87º result in a tibiofemoral angle of almost six degrees of valgus, known as the physiological valgus of the knee [7, 8]. Allen et al. looked at 210 knees between 10 and 22 years after total meniscectomy and found worsening osteoarthritis in those with leg malalignment [9]. Although patients who underwent lateral meniscectomy fared worse than those who underwent medial meniscectomy; the authors identified a subset of medial meniscectomy patients with a varus deformity who performed very badly.

The aim of our study was to assess coronal knee alignment in relation to radiological osteoarthritis grading, clinical range of movement and patient reported outcome measures 40 years after open total meniscectomies performed in adolescents.

## METHODS

Thirty eight knees (30 patients) with no other knee pathology that underwent open total meniscectomy prior to the age of 19 by a single surgeon in Tayside (Scotland) using a single operative technique were followed-up and assessed. Local ethical committee approval was obtained and all study subjects gave informed consent for clinical procedures and radiographic assessment. All patients were contacted and invited to return to clinic for a clinical and radiological assessment.

The recorded clinical data included height, weight and range of motion (ROM); measurements in both knees were assessed by one assessor (I.P.). Active knee flexion and extension ROM was measured in the supine patient with the axis of a long levered goniometer placed over the lateral femoral condyle and aligned with the greater trochanter proximally and the lateral malleolus distally. During knee extension, the patients were asked to maximally extend their knee and the heel was placed on an elevated block to allow clearance of the thigh and calf. During knee flexion, they were asked to maximally flex the hip and knee and draw the heel toward the buttocks. The average of three measurements for flexion and extension was recorded.

All patients underwent bilateral knee tibiofemoral (TF) radiological evaluation in a weight bearing anteroposterior manner (AP) with the knee flexed at 15°. This was achieved by placing the weight bearing foot’s first metatarsophalangeal joint (MTPJ) straight and at 90° with the cassette plate and the patella centralised over the femur. The beam was aimed at 2cm below the lower pole (apex) of the patella utilising fluoroscopy. The cassette used for this image was 30x40cm and the image was coned to include the distal third of the femur and the proximal third of the tibia. This knee standardisation has been found to produce an accurate and reproducible measurement of joint space width [10-12]. The tibiofemoral angles were measured using a previously described method [13] by identifying, at multiple levels, the outer cortices of both the femur and tibia and a best fit line drawn through their mid-points to provide lines that give the tibiofemoral angle at their intersection. Valgus angles were designated as positive while varus angles were designated negative. The magnitude of malalignment was calculated as the deviation from the perceived normal of 6º of valgus. Magnitude of malalignment by its very nature was always given as a positive value regardless of direction from the physiological valgus angle. All AP knee radiographs were evaluated with Ahlback [14], and Kellgren and Laurence [15] grading systems for osteoarthritis.

patient completed the KOOS (Knee Osteoarthritis Outcomes Score) [16, 17] and IKDC 2000 (International Knee Documentation Committee) [18] questionnaires in clinic. One patient’s subjective outcomes were completed at a telephone interview. Statistics were performed using SPSS (SPSS v.17) with non-parametric tests used for comparisons between variable (Wilcoxon sign rank test for paired variables and Wilcoxon two sample test for unpaired samples). Correlations were performed using Kendall Tau correlation.

## RESULTS

The sex ratio was 1: 6.5 in favour of males. Mean age at the time of meniscectomy was 15.7 (+4.9) years and at the time of follow-up, it was 56.8 (+4.9) years. The mean follow up was 41.3 (+1.2) years. Of the 38 knees, 15 underwent medial meniscectomy (MM), 15 underwent lateral meniscectomy (LM) and 8 underwent both medial and lateral meniscectomies (MLM). The body mass index (BMI) was calculated from the height and weight. BMI was 28.3 (+3.4) kg/m^2^ with no difference between the three groups.

The mean tibiofemoral angle was 3.2º (+4.8º). The mean angle for the MM group (-0.5º+4.5º) was significantly lower (i.e. more varus) compared to the LM group (5.5º+2.9º) (p=0.0004), and the MLM group (5.9º+3.9º) (p=0.003). This difference persisted when the magnitude of malalignment was compared. Magnitude of malalignment in MM (6.5º+4.5º) was significantly higher than for LM group (2.3º+1.8º) (p=0.002), and the MLM group (3.0º+2.0º) (p=0.045). Range of flexion was significantly lower in the MLM group (120.6º+6.2º) when compared to the MM group (125.0º+12.5º) (p=0.016), and the LM group (129.3º+9.8º) (p=0.026). Full descriptive statistics are included in Table **1**.

Eight patients from all three groups who underwent bilateral meniscectomies were compared with the remaining 22 patients who underwent surgery to one knee alone. The IKDC score was significantly lower in the 8 bilateral patients (50.4 *vs.* 62.8, p=0.009) when compared with the unilateral patients. There was also a significantly lower quality of life (QoL) KOOS score for the bilateral patients (47.7 *vs.* 72.0, p=0.045), but not for the remaining KOOS categories of symptoms, pain, activities of daily living (ADL) and sports. No difference in BMI was observed between the groups (27.1 kg/m^2^ *vs.* 28.7 kg/m^2^, p=0.54).

**Fig. (1):**
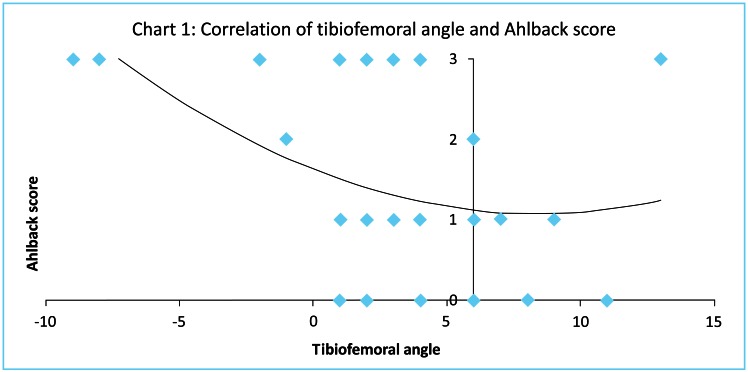
Tibiofemoral angle inversely correlated with Ahlback scores (T= -0.299 p=0.046, Kendall tau correlation).

**Fig. (2):**
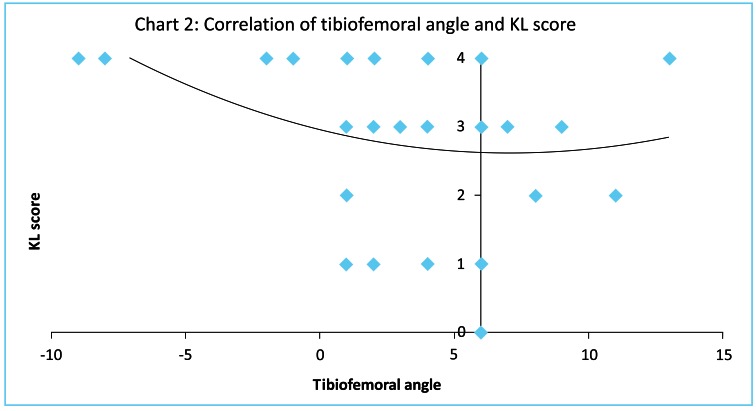
Tibiofemoral angle inversely correlated with Kellgren and Laurence (Fig. **2**) scores (T= -0.307 p=0.043, Kendall tau correlation).

Tibiofemoral angle inversely correlated with Ahlback (Fig. **1**), and Kellgren and Laurence (Fig. **2**) scores (T= -0.299 p=0.046 and T= -0.307 p=0.043 respectively, Kendall tau correlation). Magnitude of malalignment also correlated with both Ahlback, and Kellgren and Laurence scores (T=0.321 p=0.034 and T= 0.330 p=0.029 respectively, Kendall tau correlation). Range of flexion showed a strong inverse correlation with Ahlback, and Kellgren and Laurence scores (T= -0.614 p=0.00006 and T= -0.575 p=0.0002 respectively, Kendall tau correlation). Although hyperextension correlated with Ahlback score, the moderate correlation with Kellgren and Laurence score was not significant (T= -0.349 p=0.027 and T= -0.255 p=0.104 respectively, Kendall tau correlation). Radiological measures of osteoarthritis correlated poorly (p>0.05) with all the IKDC scores and KOOS. Range of motion did not correlate with tibiofemoral angle.

## DISCUSSION

The meniscus increases the tibiofemoral joint congruity by increasing the contact areas from 6-12cm^2^ to 11.60-20.13cm^2^ [19]. A 70Kg person exerts 1-2MPa of stress on the tibiofemoral joint of with menisci intact, and up to 5MPa with the menisci removed. In addition to the lack of meniscal tissue, contact stresses can also be affected by joint malalignment. A varus malalignment of 5º can change the distribution of load between the medial and lateral compartments from 70%:30% in a normally aligned knee to 90%:10% [20-22]. Medial tibiofemoral contact pressure increase of 106% and lateral compartment decrease of 89% was seen with 30º varus malalignment [23]. The maximal joint pressure centre shifts as the centre of gravity changes that may even be producing ‘condylar lift off’ during walking [24]. Increased dynamic loads in the medial compartment due to varus malalignment in osteoarthritis were found to aggravate the condition and posed the question of whether malalignment precedes or follows the onset of the disease [25].

Medial meniscectomy in an already varus knee has demonstrated a higher risk of osteoarthritis than in a normally aligned one [9]. A small degree of varus malalignment was found to cause dramatic alteration in articular surface contact pressure, especially in the presence of chondral damage or medial meniscectomy [26], where medial meniscectomy equated to 1.5-2º of loss in anatomic valgus alignment and contributed to radiographic loss of medial joint space. As the rate of osteoarthritis progression observed seemed to be similar in mild and moderate varus malalignment, it was postulated that articular cartilage in the absence of a ‘breech’ may be able to tolerate the changes in load distribution following the removal of a meniscus. Medial meniscectomy in knees with <4º of anatomic valgus seemed to do worse following medial meniscectomy [27] and was observed as the only significant factor for the development of degenerative changes post meniscectomy [28]. In our cohort there was a significant difference in the tibiofemoral angle and the magnitude of malalignment between the three meniscectomy groups. The medial meniscectomy group fared worse, with more varus malalignment. There was also good correlation between the malalignment and the radiographic measures of osteoarthritis in keeping with previous studies.

Previously it was observed at 30 year follow-up that double meniscectomies fared worse Tapper & Hoover and WOMAC scores than lateral meniscectomies, and lateral worse than medial meniscectomies [29]. These findings were consistent with some [9, 30-32], but not all [33] earlier studies. We believe that the Tapper and Hoover system was used incorrectly in the earlier study by grouping together excellent and good [35]. The WOMAC score used, although reliable in measuring knee disability and more responsive than the SF-36 [36, 37], has been superseded by the KOOS, a system incorporating WOMAC [16]. Our cohort, reporting the longest reported follow-up in literature, failed to show a difference in patient reported outcome measures (PROMs) between the types of meniscectomy in a single knee but did show worse IKDC and KOOS QoL scores for those who underwent bilateral meniscectomy suggesting that it is actually the burden of disease rather than the type of meniscectomy that affects PROMs. The PROMs used in our study have been shown to be valid, reliable and responsive, as well as acceptable for patients [34].

Poorer radiological outcomes following lateral as opposed to medial meniscectomy have been reported by most [9, 29, 38, 39] but not all [33, 40] previous studies. These findings are supported by in vitro studies showing 70% load transmission through the lateral meniscus as opposed to 50% through the medial side [41], and the relatively incongruent lateral tibiofemoral joint. More forces are however transmitted through the medial tibiofemoral joint [42] due to the more concave and congruent medial tibial condyle providing a 1.6 times greater contact area [20]. This is supported by the fact that the medial tibia has relatively stronger bone [43-45]. We can explain our findings by deducing that even though greater forces are transmitted through the medial compartment, the contact stresses may not be significantly different due to the increase congruency and contact area of the medial joint. Therefore in our long term study, the effects of the site of total meniscectomy, medial, lateral or indeed both, did not demonstrate a significant difference in the degree of ultimate radiographic tibiofemoral osteoarthritis grade. This finding is supported in other open total meniscectomy studies [46].

All scoring systems have their limitations. The radiological scoring system used considers loss of joint height, osteophytes, cysts and other radiological changes that could influence pain and loss of function. Only asymmetrical loss of joint height would affect malalignment, whereas the other radiological factors would also affect disability. Having said this it is still unclear if the resultant malalignment is simply due to the removal of the meniscus with an assumed 1-2º [26] of resultant malalignment, the change in force distribution across the TFJ, the progression of ensuing OA or a combination of all the above. A limitation was the lack of a healthy age, gender and race matched control group to compare outcomes. Other potential pitfalls mentioned in previous studies [47] concerning the standardisation of radiographs for evaluation were taken into consideration when planning for the study and were avoided.

## CONCLUSION

Forty years following adolescent open total meniscectomies, there was significantly more varus deformity and malalignment after medial meniscectomies. Tibiofemoral angle and malalignment correlated with worsening radiographic scores of osteoarthritis. Range of flexion was reduced significantly in those who underwent both medial and lateral meniscectomies of the same knee. Patients who underwent bilateral meniscectomies had significantly worse IKDC score and quality of life KOOS. While malalignment and reduced range of movement correlate well with worsening radiographic signs of arthritis, patient reported outcome measures do not.

## Figures and Tables

**Table 1:** Full descriptive statistics for the medial meniscectomy (MM), lateral meniscectomy (LM), and both medial and lateral meniscectomies (MLM) groups as well as for all patients.

	n=	MM	LM	MLM	All
Male: Female	30	10: 2	11: 0	5: 2	26: 4
Age at FU (Mean +/- SD)	30	57.3+3.9	55.5+5.4	54.1*+*6.3	55.9+5.1
Type of meniscectomy (bilateral knees)		15 (3)	15 (4)	8 (1)	38 (8)
BMI (Mean +/- SD) in kg/m^2^	30	28.2+3.6	28.0+2.7	29.0+4.3	28.3+3.4
IKDC score (Mean +/- SD)	30	58.8+9.7	61.7+14.4	56.3+12.6	59.4 +12.0
KOOS	30				
- symptoms		68.3+20.8	60.7+24.4	57.7+20.5	63.2+22.0
- Pain		73.7+21.3	70.2+20.5	75.8+21.6	72.6+20.6
- Activities of Daily Living (ADL)		75.7+19.8	78.5+19.4	80.3+22.0	77.7+19.6
- Sports		61.0+27.7	54.0+31.6	72.1+27.1	60.3+28.8
- Quality of Life (QoL)		62.9+26.6	53.3+28.8	75.9+23.5	61.5+27.5
Valgus angle (Mean +/- SD)	38	-0.5+4.5 *	5.5+2.9	5.9+3.9	3.2+4.8
magnitude of malalignment (Mean +/- SD)	38	6.5+4.5 *	2.3+1.8	3.0+2.0	4.1+3.7
Flexion (Mean +/- SD)	38	125.0+12.5	129.3+9.8	120.6+6.2 *	127.1+10.7
Hyperextension (Mean +/- SD)	38	-6.3+5.2	-3.7+5.2	-5.0+8.9	-5.0+6.0

* Significantly differs at p<0.05
